# Spinal Cord Injury Reveals Multilineage Differentiation of Ependymal Cells 

**DOI:** 10.1371/journal.pbio.0060182

**Published:** 2008-07-22

**Authors:** Konstantinos Meletis, Fanie Barnabé-Heider, Marie Carlén, Emma Evergren, Nikolay Tomilin, Oleg Shupliakov, Jonas Frisén

**Affiliations:** 1 Department of Cell and Molecular Biology, Karolinska Institute, Stockholm, Sweden; 2 Department of Neuroscience, Karolinska Institute, Stockholm, Sweden; Columbia University, United States of America

## Abstract

Spinal cord injury often results in permanent functional impairment. Neural stem cells present in the adult spinal cord can be expanded in vitro and improve recovery when transplanted to the injured spinal cord, demonstrating the presence of cells that can promote regeneration but that normally fail to do so efficiently. Using genetic fate mapping, we show that close to all in vitro neural stem cell potential in the adult spinal cord resides within the population of ependymal cells lining the central canal. These cells are recruited by spinal cord injury and produce not only scar-forming glial cells, but also, to a lesser degree, oligodendrocytes. Modulating the fate of ependymal progeny after spinal cord injury may offer an alternative to cell transplantation for cell replacement therapies in spinal cord injury.

## Author Summary

Spinal cord injuries occur in more than 30.000 individuals each year worldwide and result in significant morbidity, with patients requiring long physical and medical care. The recent identification of resident stem cells in the adult spinal cord has opened up for the possibility of pharmacological manipulation of these cells to produce cell types promoting recovery after injury. We have employed genetic tools to specifically address the identity and reaction to injury of a spinal cord subpopulation of cells known as ependymal cell. Genetic labeling of this putative stem cell population allows for the evaluation of stem cell activity in vitro and in vivo. We found that ependymal cells lining the central canal act as neural stem cells in vitro and contribute extensively to the glial scar in vivo. Interestingly, injury induces proliferation of ependymal cells and migration of ependyma-derived progeny towards the site of injury. Moreover, ependymal cell progeny differentiate and give rise to astrocytes as well as myelinating oligodendrocytes. In summary, our results point to ependymal cells as an attractive candidate population for non-invasive manipulation after injury.

## Abstract 3

Spinal cord injury induces the proliferation of putative stem cells derived from the ependyma that migrate towards the site of injury. Ependymal cell progeny differentiate and give rise to astrocytes as well as myelinating oligodendrocytes.

## Introduction

Transplantation of different types of stem cells improves functional recovery after spinal cord injury in rodents and primates. The beneficial effects appear to be mediated by several mechanisms, including replacement of lost cells, secretion of neurotrophic factors, and probably most importantly, the generation of oligodendrocytes that remyelinate spared axons in the vicinity of a lesion [1,2].

Neural stem cells present in the adult spinal cord can be propagated in vitro [3,4], and promote functional recovery when transplanted to the injured spinal cord [5]. Endogenous neural stem cells could therefore be attractive candidates to manipulate for the production of desired progeny after spinal cord injury as an alternative to stem cell transplantation. This approach would offer a noninvasive strategy that avoids the need for immune suppression, but has been held back by difficulties in identifying adult spinal cord neural stem cells and developing rational ways to modulate their response to injury. Studies using indirect techniques have suggested that the neural stem cell potential in the adult rodent spinal cord resides in the white matter parenchyma [6,7] or close to the central canal, either in the ependymal layer [8] or subependymally [9].

We have employed genetic fate mapping to characterize a candidate neural stem cell population in the adult spinal cord and show that close to all in vitro neural stem cell potential resides within the population of ependymal cells. Ependymal cells give rise to a substantial proportion of scar-forming astrocytes as well as to some myelinating oligodendrocytes after spinal cord injury. Modulating the fate of ependymal cell progeny after injury could potentially promote the generation of cell types that may facilitate recovery after spinal cord injury.

## Results

### Genetic Labeling of Cells in the Adult Spinal Cord Ependymal Layer

In order to fate map candidate neural stem cells close to the central canal, we generated two transgenic mouse lines expressing tamoxifen-dependent Cre recombinase (CreER) under the control of *FoxJ1* (HFH4) or *Nestin* regulatory sequences. FoxJ1 expression is specific to cells possessing motile cilia or flagella [10–13]. In the adult forebrain, a subset of astrocytes in the subventricular zone contact the ventricle and have an immotile primary cilium [14], but FoxJ1 expression is restricted to cells with motile cilia [10–13]. Nestin is expressed in central nervous system stem and progenitor cells during development and in adulthood [15–19]. In the adult spinal cord, nestin is expressed by cells lining the central canal, endothelial cells, and sparse white matter glial cells [20]. The second intron enhancer in the *Nestin* gene allows for selective expression of CreER in the neural lineage [21], eliminating expression in for example endothelial cells.

**Figure 1:**
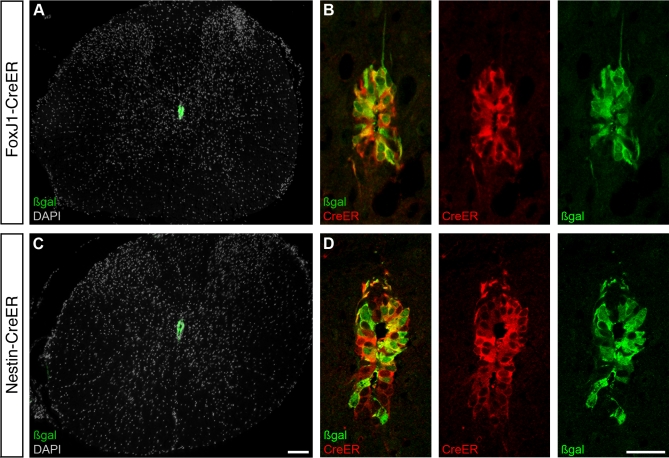
Genetic Labeling of Spinal Cord Ependymal Cells Transgenic mice with tamoxifen-inducible Cre recombinase (CreER) under the control of the *FoxJ1* promoter (A and B) or the *Nestin* second intron enhancer (C and D) drive expression and induce recombination after 5 daily tamoxifen injections (resulting in β-gal expression) in cells lining the central canal in the adult spinal cord. (A and C) Overviews of coronal sections from the thoracic spinal cord and (B and D) higher magnification of the central canal region demonstrating recombination in the majority of cells and cytoplasmic CreER protein 6 days after the last tamoxifen administration. Cell nuclei are visualized with DAPI in (A and C). Scale bars indicate 100 μm in (A) and (C), and 25 μm in (B) and (D).

CreER expression in the adult spinal cord is limited to cells lining the central canal in both the *FoxJ1-CreER* and *Nestin-CreER* mouse lines (Figure 1). Administration of tamoxifen to mice on an R26R [22] or Z/EG [23] Cre reporter background allows inducible, permanent. and heritable genetic labeling by the expression of β-galactosidase (β-gal; R26R) or GFP (Z/EG) in cells expressing CreER (the strategy is schematically depicted in Figure S1). Recombination in the absence of tamoxifen was exceptionally rare (<1 cell/30 coronal 20-μm-thick sections in both transgenic lines) and limited to CreER-expressing cells in the ependymal layer. Administration of tamoxifen (five daily injections) resulted in recombination of the reporter allele (Figure 1A–1D) in 82 ± 4% of transgene-expressing cells in *Nestin-CreER* mice and 88 ± 4% in *FoxJ1-CreER* mice (mean ± standard deviation [SD], *n* = 6 mice for each mouse line).

### Phenotypic Characterization of Adult Spinal Cord Ependymal Cells

The cells at the central canal expressing CreER protein from the *Nestin-CreER* or *FoxJ1-CreER* transgene are immunoreactive to Crocc, a marker for ciliated cells (Figure S2). They contain the intermediate filaments nestin and vimentin, associated with immature neural cells [15], but notably not glial fibrillary acidic protein (GFAP) (Figures S2 and S3), which is present in some neural stem cells in the adult forebrain [24]. The transgene expressing cells display other markers associated with neural stem/progenitor cells such as CD133/prominin-1, Musashi1, PDGFR-α, Sox2, Sox3, and Sox9 but are negative for the oligodendroglial progenitor marker Olig2 (Figures S2 and S3). All above-mentioned proteins appear uniformly expressed by the cells lining the central canal, and we have not found any molecular marker delineating any subpopulations.

**Figure 2:**
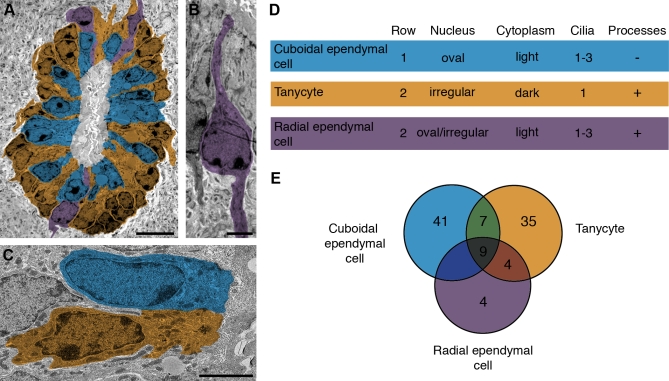
Characterization of Spinal Cord Ependymal Cells (A–C) Immunoelectron microscopy of the central canal in a *FoxJ1-CreER* mouse. Pseudocoloring in (A) illustrates the localization of CreER-immunoreactive radial ependymal cells (B), cuboidal ependymal cells, and tanycytes (C). (D) Table with color code for (A–C), describing the characteristics of the three cell types that line the central canal. (E) Venn diagram illustrating the percentage of cuboidal ependymal cells, tanycytes, radial ependymal cells, and intermediate morphologies (see Figures S4 and S5, and Table S1 for details on the ultrastructural analysis). Scale bars indicate 10 μm in (A) and 3 μm in (B) and (C).

Immunoelectron microscopy established that the *Nestin-CreER* and *FoxJ1-CreER* transgenes are expressed in identical cell populations by the central canal; their expression is restricted to lumen-contacting cells with motile cilia (9 + 2 axonemes), and all such cells express both transgenes (Figures 2A–2C, S4, and S5). Ultrastructural analysis in serial sections revealed morphological heterogeneity among the lumen-contacting ciliated cells, with some cells displaying typical cuboidal ependymal cell morphology and others a tanycyte morphology [25] (Figures 2C, 2D, S4, and S5). In addition, there is a less numerous third cell type, which we refer to as a radial ependymal cell. Radial ependymal cells share the morphology of the cytoplasm, and often nucleus, with ependymal cells, but have a long basal process (Figures 2B, 2D, and S5). The radial ependymal cells almost invariably reside in the dorsal or ventral pole of the ependymal layer, with a basal process oriented along the dorsoventral axis (Figures 2A and S5). Although the lumen-contacting ciliated cells can be subdivided into these three groups, cells with intermediary phenotypes are frequent (Figure 2E and Table S1), which together with their homogeneous molecular profile suggests that they are closely related. The naming of ependymal cell types is based solely on morphological criteria and does not imply any function. The central canal-contacting ciliated cells have in common that they reside in the ependymal layer, thus we collectively refer to them as ependymal cells.

### Adult Spinal Cord Stem Cells Are Largely Contained within the Ependymal Cell Population

**Figure 3:**
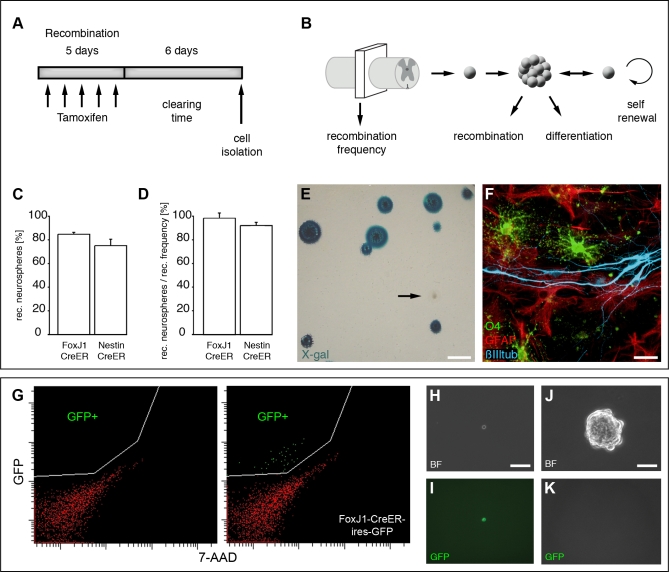
Ependymal Cells Display Neural Stem Cell Properties In Vitro (A) Schematic depiction of tamoxifen administration paradigm and (B) analysis of neural stem cell properties and in vivo and in vitro recombination frequency. (C) The high proportion of recombined (rec.) neurospheres (mean + SD, *n* = 6 mice for each transgenic mouse line) demonstrates that the majority derives from ependymal cells. (D) Estimate of the proportion of neurospheres that derive from ependymal cells by normalization to the recombination rate in tissue sections of the spinal cords that were used to initiate the cultures (mean ± SD). (E) Recombined primary neurospheres from *FoxJ1-CreER* mice on R26R background visualized by X-gal staining (arrow points to one unrecombined neurosphere). (F) Differentiation of a clonally derived recombined neurosphere into neurons (βIIItub), astrocytes (GFAP), and oligodendrocytes (O4). (G) Flow cytometric isolation of GFP^+^ cells from the spinal cord of adult *FoxJ1-CreER* mice based on the IRES-GFP signal (GFP gate). 7-AAD labels dead cells, which were excluded. (H–K) Brightfield (BF) and fluorescent images (I and K) of a single GFP^+^ sorted cell and a neurosphere (J) formed from such a cell, which is GFP^−^ due to the lack of FoxJ1 expression (K). Scale bars indicate 400 μm in (E), 20 μm in (F), 100 μm in (H) and (I), and 50 μm in (J) and (K).

Adult spinal cord neural stem cells can be propagated in vitro [3], but their precise identity has been difficult to establish unequivocally [6–9]. We utilized our genetic labeling paradigms to ask whether adult spinal cord ependymal cells have neural stem cell properties in vitro. Adult *FoxJ1-CreER* and *Nestin-CreER* mice on Cre recombination reporter background (R26R or Z/EG) received five daily injections of tamoxifen to induce recombination, and primary cultures were initiated after an additional 6 d without tamoxifen (Figure 3A). Tamoxifen and its active metabolite 4-hydroxytamoxifen have a half-life of 6–12 h in the mouse [26], and accordingly CreER protein was no longer detectable in the nucleus after 6 d without tamoxifen (Figure 1B and 1D). Spinal cords were dissociated and plated at clonal density in standard conditions that allow for neurosphere formation (Figure 3B). We found that 76 ± 5.7% of neurospheres from *Nestin-CreER* and 85 ± 2.2% from *FoxJ1-CreER* mice were recombined and thus derived from recombined ependymal cells (mean ± SD, *n* = 6 mice analyzed separately per line, Figure 3C and 3E). Neurospheres were either homogeneously recombined or not recombined, verifying their clonal origin.

Since recombination never was fully penetrant in the ependymal cells, the analysis of the proportion of neurospheres that were recombined may underestimate the true contribution of this cell population to neurosphere formation. If there is a stochastic distribution of recombination within the CreER expressing cell population, rather than recombination demarcating a subpopulation that differs with regard to neurosphere-forming potential, one can estimate the contribution of the cell population to neurosphere formation by normalizing it to the observed recombination rate. To estimate the theoretically maximal proportion of neurosphere-initiating cells that are ependymal cells, we analyzed the recombination frequency in CreER-expressing cells in sections from each spinal cord sample that was used for neurosphere cultures (Figure 3B). Normalizing the recombination frequency in neurospheres to the recombination frequency in the CreER-expressing cells in vivo, suggested that close to all neurosphere-initiating potential could reside within the ependymal cell population under these conditions (Figure 3D).

Progenitor cells with limited self-renewal capacity can give rise to neurospheres, but are incapable of generating new neurospheres when passaged more than twice [27,28]. We found that 100% of the recombined neurospheres from both *Nestin-CreER* and *FoxJ1-CreER* could be serially passaged at least eight times to give rise to new neurospheres (*n* = 6 neurospheres per 4 transgenic mice). The number of cells increased exponentially during passaging (Figure S6). Analysis of the differentiation potential of ependymal cell-derived neurospheres after three passages revealed that 100% of the neurosphere clones were multipotent and differentiated into neurons, astrocytes, and oligodendrocytes (Figure 3F).

We also isolated prospectively identified ependymal cells by flow cytometry independently of Cre-mediated recombination by utilizing the green fluorescent protein (GFP) expression under the bicistronic control of the FoxJ1 promoter (Figures 3G and S1). Flow cytometric isolation of adult spinal cord cells substantially reduced neurosphere formation, and 0.18 ± 0.06% (mean ± SD from in average 1,600 GFP-positive (GFP^+^) cells/mouse, *n* = 6 mice analyzed separately) of GFP^+^ ependymal cells formed neurospheres (Figure 3H–3K). In contrast, not a single neurosphere developed from the same number of cells in the GFP^−^ non-ependymal fraction from any animal in the same experiments. Thus, the neural stem cell potential in the adult spinal cord, at least under the conditions employed here, largely resides within the ependymal cell population.

### Ependymal Cells Self-Renew In Vivo

**Figure 4:**
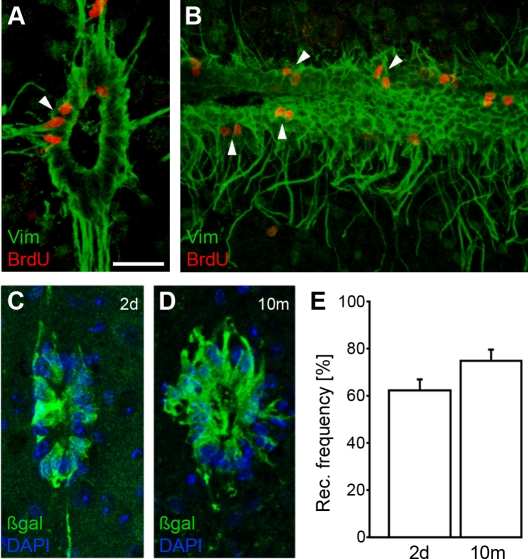
Ependymal Cells Self-Renew In Vivo (A and B) BrdU incorporation in ependymal cells after 4-wk administration in the drinking water. Many of the labeled cells are found in pairs (arrowheads). (A) shows a coronal and (B) a sagittal section. (C–E) The recombination (rec.) rate of ependymal cells remains at the same level from 2 d to 10 mo after tamoxifen administration (mean and SD from 3–4 mice at each time point). The scale bar indicates 25 μm in (A–D).

Cells in the adult spinal cord ependymal layer proliferate, albeit slowly or rarely [8]. Continuous administration for one month in the drinking water of 5-bromo-2-deoxyuridine (BrdU), which is incorporated into DNA in cells in S-phase, resulted in labeling of 19.9 ± 4.2% of ependymal cells (mean ± SD from three mice, Figure 4A and 4B). The BrdU-labeled ependymal cells constituted 4.8 ± 0.9% of all BrdU-labeled cells in a spinal cord segment (mean ± SD from three mice). The majority of BrdU-labeled ependymal cells were found in pairs, indicating that most mitoses resulted in self-duplication rather than the generation of another cell that had left the ependymal layer (Figure 4A and 4B). In line with this, analysis of the distribution of recombined cells up to 8 mo after tamoxifen administration in the *FoxJ1-CreER* and *Nestin-CreER* mice did not provide evidence for the generation of cells that leave the ependymal layer under normal conditions (unpublished data).

Whether a specific cell population is derived from another cell type or it is maintained through self-duplication can be established by analyzing the genetic labeling frequency at different time points after induction of recombination [29,30]. There was no reduction in the proportion of recombined ependymal cells for up to 10 mo after tamoxifen administration (Figure 4C–4E), indicating that ependymal cells are maintained by self-renewal and are not replenished by another cell population.

### Ependymal Cells Are Activated by Spinal Cord Injury

**Figure 5:**
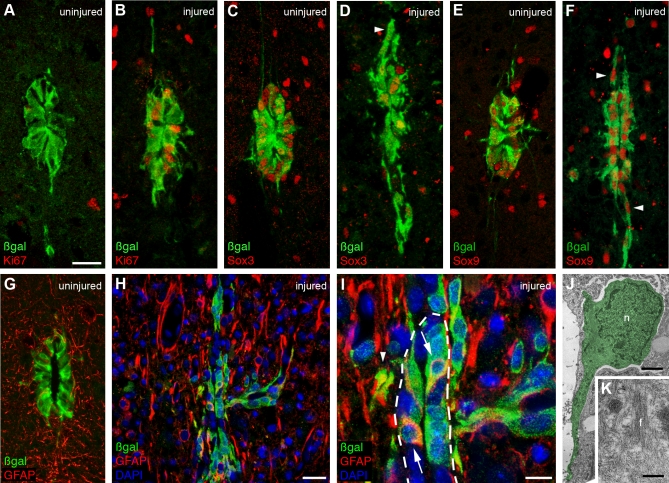
Ependymal Cells Are Activated by Injury Uninjured and adjacent injured segments from mice 4 d after a dorsal funiculus incision. Recombined cells leave the ependymal layer in the injured segments (arrowheads). (A and B) Ki67 immunoreactivity indicates ependymal proliferation in the injured, but not in the uninjured segment. Migrating recombined cells lose Sox3 expression (D), but most are Sox9 immunoreactive, and a smaller population is GFAP immunoreactive (F–I). Some ependymal cells within the ependymal layer (outlined by hatched line in [I]) become GFAP immunoreactive (arrows). (J and K) Electron micrographs of an extended ependymal cell with a dense filamentous matrix (f) in the cytoplasm 4 wk after injury in a *FoxJ1-CreER* mouse. n, nucleus. Scale bars indicate 25 μm in (A–H), 10 μm in (I), 1.5 μm in (J), and 0.2 μm in (K).

We next assessed the response of ependymal cells to spinal cord injury. We used the same labeling paradigm as before (Figure 3A), with a 6-d period between the last tamoxifen dose and the injury. This ensures that all recombination occurs prior to the insult and that even if other cells than ependymal cells would start to express the *FoxJ1-CreER* or *Nestin-CreER* transgene in response to the injury (nestin is indeed expressed by reactive astrocytes [20]), it would not result in recombination. An incision in the dorsal funiculus, which does not compromise the integrity of the ependymal layer, dramatically increased the proliferation of ependymal cells (Figures 5A, 5B, and S7). In contrast to the uninjured spinal cord, where proliferation of ependymal cells appears largely limited to self-renewing divisions, recombined cells started to migrate and were located outside the ependymal layer 4 d after the injury (Figure 5C–5I). Migrating recombined cells lost their ependymal phenotype as judged by the loss of immunoreactivity to Sox2 and Sox3 and lack of CreER expression from the *FoxJ1* promoter (Figure 3D and unpublished data). Most emigrating cells expressed Sox9 and some the astrocyte marker GFAP (Figure 5F, 5H, and 5I). Ultrastructural analysis revealed that ependymal cell morphology was largely unaltered by the injury, with the exception of a darker cytoplasm due to a higher content of filaments (Figure 5J and 5K).

### Ependymal Cells Contribute to Scar Formation after Injury

**Figure 6:**
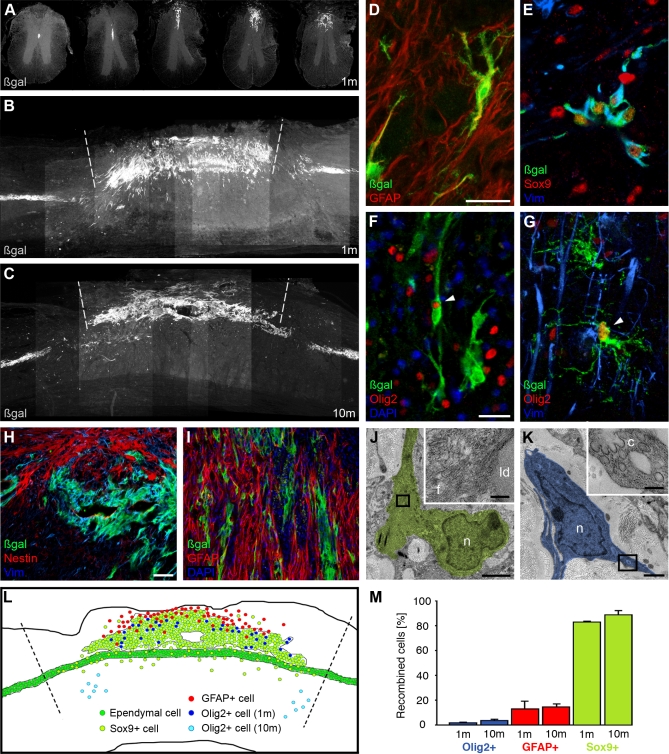
Ependymal Cells Contribute to Scar Formation after Spinal Cord Injury (A) Distribution of β-gal–immunoreactive ependyma-derived cells in coronal sections from an uninjured segment (left) further towards the lesion epicenter (right). (B and C) Sagittal sections show the distribution of recombined cells 1 mo (B) and 10 mo (C) after a dorsal funiculus incision (indicated by hatched lines). (D and E) Recombined cells outside the ependymal layer display either (D) the astrocytic marker GFAP or (E) a Sox9/vimentin double-positive profile. (F and G) Other recombined cells are Olig2 immunoreactive (arrowheads) and have oligodendrocyte morphology at later time points ([F] is at 1 mo and [G] 10 mo). (H and I) The scar tissue is compartmentalized with patches of ependyma-derived cells. (J and K) Electron micrographs of β-gal–immunoreactive cells with astrocyte (J) or oligodendrocyte (K) morphology. Boxed areas are shown at a higher magnification in the insets. (L) Drawing (based on [C]) depicting the distribution of recombined cells of different phenotypes. (M) Marker profile of recombined ependyma-derived cells (mean and SD from 3 mice at each time point) in the area encompassing the injury indicated by hatched lines in (L). c, caveolae; f, filaments; ld, lipid droplet; n, nucleus. Scale bars indicate 25 μm in (D–G) and (H), 50 μm in (I), 2 μm in (J), 1 μm in (K), and 0.15 μm in insets in (J) and (K).

Ependymal progeny migrated towards the injury site in the dorsal funiculus and an increasing number of recombined cells accumulated in the forming glial scar over several weeks and remained there for at least 10 mo after the insult (Figure 6A–6C). The recombined ependyma-derived cells occupied 18.3 ± 6.9% (mean ± SD from three *FoxJ1-CreER* mice) of the area in the scar tissue 2 wk after the injury, which is likely to be a slight underestimate of the true contribution since recombination never was fully penetrant. The ependyma-derived cells were not evenly distributed throughout the injury site, but the scar consisted of patches of recombined and unrecombined cells (Figure 6H and 6I). The reaction of the ependymal cells was restricted to the injured segment and was absent in adjacent segments (Figures 5A–5H and 6A–6C), which are indirectly affected by the severance of axons and Wallerian degeneration. There were no recombined cells outside the ependymal layer in animals in which only the spinal cord was exposed but no lesion was made (sham lesion, Figure S8), and a lesion did not induce recombination in animals that had not received tamoxifen (unpublished data).

Since some ependymal cells extend processes along the dorsolateral midline, it was possible that the activation of ependymal cells by a dorsal funiculus incision was triggered by the severance of such processes. To investigate this, we performed incisions in the lateral spinal cord, which do not directly injure the ependymal cell processes in the dorsolateral midline. In these animals, ependymal cell progeny were generated and migrated laterally towards the injury (Figure S8). The ependyma-derived cells migrating to the lesion appeared less numerous after a lateral than after a dorsal incision, suggesting that severance of ependymal cell processes in the midline is not necessary for the activation of ependymal cells, but that it may augment their reaction. The migration of ependyma-derived cells to the site of injury suggests the presence of attractive signals originating in the lesion area. SDF1, through its receptor CXCR4, mediates attraction of progeny from neural stem/progenitor cells after some types of injuries [31,32]. The majority of ependymal cells as well as their progeny were, however, negative for CXCR4 (Figure S9), making it unlikely that this receptor mediates the attraction of ependymal cell progeny to a spinal cord lesion.

Analysis of the fate of the ependymal cell progeny by molecular markers and electron microscopy after a dorsal funiculus incision revealed that the majority were immunoreactive to Sox9 and vimentin and had an astrocyte-like morphology (Figures 6E, 6H, 6J, 6M, and S10). A smaller subpopulation of the recombined cells expressed GFAP and nestin, but the vast majority of cells with this phenotype were not recombined (Figure 6D, 6H, 6I, and 6M). Recombined GFAP- and nestin-expressing cells were typically located close to the surface of the spinal cord (Figure 6D, 6H, 6I, and 6L), whereas the Sox9- and vimentin-expressing cells were most abundant in the core of the scar tissue (Figures 6L and S10). We conclude that the glial scar is comprised of two different populations of astrocyte-like cells, where the majority of the Sox9^+^/vimentin^+^ population derives from ependymal cells and the GFAP^+^/nestin^+^ cells are mainly reactive resident astrocytes.

We further investigated the contribution of ependymal cells to other lineages. None of the recombined cells in the scar tissue had neuronal morphology or were immunoreactive to the neuron-specific epitope NeuN (unpublished data). A population of recombined cells expressed Olig2 (Figure 6F and 6G). The first month after injury, Olig2-expressing recombined cells were scattered throughout the injury site and had an ultrastructural morphology corresponding to immature oligodendrocytes (Figure 6F, 6K, 6L, and 6M). At later time points, Olig2-expressing ependyma-derived cells were excluded from the scar tissue and were restricted to the uninjured tissue that bordered the scar (Figure 6G, 6L, and 6M). Lesions in the lateral funiculus resulted in the generation of ependymal progeny of the same fates as after a dorsal funiculus incision (Figure S8).

### Relationship between Ependymal Cell Progeny, Extracellular Matrix Molecules, and Axons in Spinal Cord Scar Tissue

**Figure 7:**
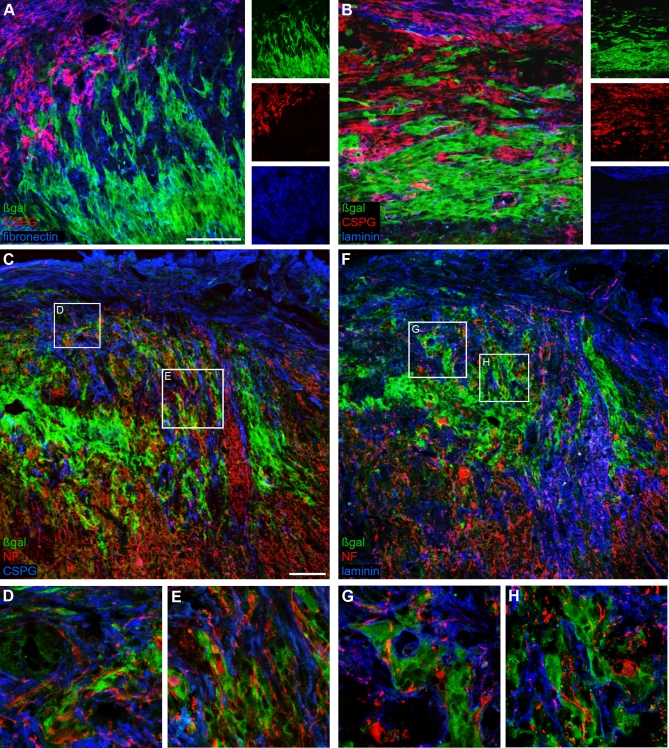
Relationship between Ependymal Cell-Derived Progeny, Axonal Growth-Modulating Molecules, and Axons in the Scar Tissue (A–E) Chondroitin sulphate proteoglycans (CSPG), which are axonal growth inhibitory, are abundant in the scar tissue 2 wk after a dorsal funiculus lesion. CSPG are present in a complementary and nonoverlapping pattern to β-gal–expressing ependyma-derived cells. The extracellular matrix proteins fibronectin (A) and laminin (B and F), which are permissive to axonal sprouting, are widely distributed within the scar tissue and overlap with β-gal–expressing ependyma-derived cells. (C–H) Neurofilament (NF)-immunoreactive axons in the scar tissue are rarely present in CSPG^+^ areas (C–E) but are associated with ependymal cell progeny (C–H) and laminin (F–H) in the core of the forming scar tissue 2 wk after injury. Scale bars indicate 100 μm.

The scar tissue that forms at spinal cord injuries is thought to inhibit axonal growth [33,34]. Chondroitin sulphate proteoglycans (CSPG) appear to be the principal axonal growth inhibiting molecules in glial scars [35]. Ependyma-derived cells at the injury formed a complementary nonoverlapping pattern with areas that were CSPG immunoreactive (Figure 7A and 7B), indicating that ependymal cell progeny do not contribute to the production of axonal growth-inhibiting CSPG.

In parallel with the production of axonal growth-inhibiting factors in the glial scar, there is an increase in some axonal growth-promoting molecules, such as the extracellular matrix molecules laminin and fibronectin [36,37]. In the injury model employed here, axons send sprouts into the scar tissue, mainly during the first month after an injury, and the axons are preferentially associated with areas in the scar tissue that have high levels of laminin [38,39]. Both laminin and fibronectin immunoreactivity were widely distributed throughout the scar tissue, overlapping both with CSPG-immunoreactive domains and areas occupied by ependyma-derived cells (Figure 7A and 7B). Neurofilament-immunoreactive axons were present in the center of the scar tissue and were often wiggly and oriented in all directions (Figure 7C–7H). This is in contrast to the rostrocaudal orientation of axons seen in the uninjured dorsal funiculus, suggesting that many of the axons present in the scar were severed and sprouting into the scar tissue [39]. Neurofilament-immunoreactive axons were present in domains dominated by ependyma-derived cells, as well as in other areas of the scar where these cells were less abundant (Figure 7C–7H). Axons were often present in direct proximity to ependyma-derived cells (Figure 7D, 7E, 7G, and 7H). The finding that ependyma-derived progeny is not associated with the main scar-associated axonal growth-inhibiting factor, CSPG, together with their proximity to axonal sprouts, argues against these cells being a major factor in glial scar-associated axonal growth inhibition.

### Ependymal Cells Generate Oligodendrocytes after Injury

**Figure 8:**
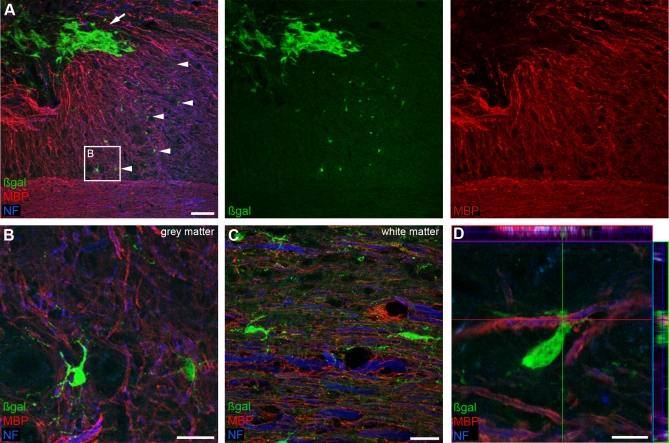
Ependymal Cells Give Rise to Oligodendrocytes after Injury (A) Ependymal cell-derived progeny are most abundant within the core of the scar tissue forming at the injury (arrow). Ependyma-derived cells are also found, more sparsely, over a larger area in the intact tissue bordering the lesion (arrowheads), where they are associated with myelin basic protein (MBP)-immunoreactive myelin ensheathing neurofilament (NF)-immunoreactive axons. (B–D) Ependymal cell-derived progeny harboring an oligodendrocytic morphology are found both in the grey (B) and white matter (C), and some recombined processes wrap around myelinated axon (D). Scale bars indicate 100 μm in (A), 25 μm in (B) and (C), and 10 μm in (D).

The finding that some ependymal cell progeny displayed a marker profile and ultrastructural morphology suggesting oligodendroglial differentiation (Figure 6) prompted us to characterize these cells further and to address whether they may contribute to axonal remyelination at later time points. Ten months after spinal cord injury, the majority of ependyma-derived progeny are located in the scar tissue that has formed at the injury site, but a substantial number of cells are sparsely distributed in a large area of the intact grey and white matter bordering the lesion (Figure 8A–8C). Most of these cells are Olig2^+^ and display mature oligodendrocyte morphology with processes that extend along and enwrap myelin basic protein (MBP)-immunoreactive myelin ensheathing axons (Figure 8B–8D).

**Figure 9:**
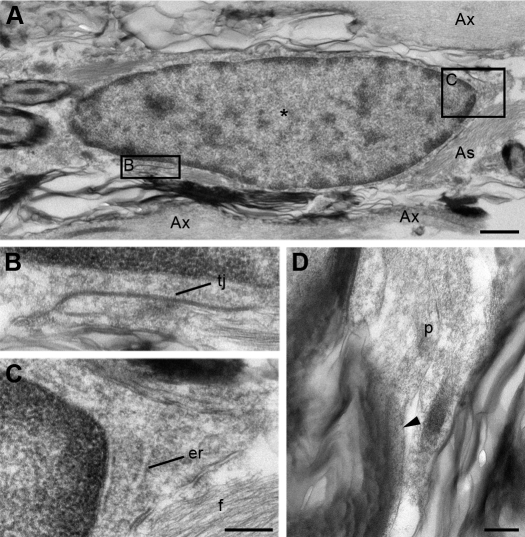
Ultrastructure of an Ependyma-Derived Oligodendrocyte Electron micrograph of an ependyma-derived β-gal–expressing cell in the nuclear plane 10 mo after injury (nucleus labeled with asterisk in [A]). (A) The cell displays ultrastructural characteristics of a mature oligodendrocyte such as a denser cytoplasm with fewer intermediate filaments than that of a neighboring astrocyte (As), (B) tight junctions (tj) between the cell body, and an oligodendrocyte process of another cell and (C) granular endoplasmic reticulum (er) cycternae in the perikaryon. Ax, axon; f, filamentous matrix. (D) A process (p) of the cell adjacent to the myelin sheath on an axon. Scale bars indicate 1 μm in (A) and 250 nm in (C) and (D).

Nuclear regions and processes of two ependyma-derived cells were followed in the electron microscope in serial utrathin sections (Figures 9 and S11, and unpublished data). They both displayed a typical mature oligodendrocyte morphology [25], such as oval nuclei with clumped chromatin, a cytoplasmic matrix that appeared denser than in surrounding astrocytes, a granular endoplasmic reticulum represented by several short cysternae, and tight junctions with adjacent oligodendrocyte processes (Figure 9). Few processes emerged from the cell body, and unlike those of astrocytes, they did not form many branches and did not contain evident fibrils (Figure 9). The processes of the recombined cells could be traced along axons, surrounding their myelin sheaths (Figure 9D). Thus, in addition to the generation of astrocytes, ependymal cells generate myelinating oligodendrocytes.

## Discussion

Stem cells are notoriously difficult to identify, and their localization in the adult spinal cord has been controversial [6–9]. We report that ependymal cells constitute the vast majority of cells displaying in vitro neural stem cell properties in the adult spinal cord. Ependymal cells self-renew in vivo, but do not generate appreciable numbers of other cell types under homeostatic conditions. Their normally limited proliferation increases dramatically after spinal cord injury and they then produce oligodendrocytes, and more abundantly, astrocytes that migrate to the site of injury and make a substantial part of the glial scar.

The immediate descendants of tissue stem cells, progenitor cells with limited self-renewal capacity and/or lineage potential, can in some situations acquire stem cell properties [40]. For example, spermatogonial progenitor cells can regain stem cell function after injury and during aging and forebrain neurospheres may be derived from committed progenitors [41,42]. It appears unlikely that this would explain the neural stem cell properties displayed by ependymal cells in vitro, as they are not replenished by any other cell type in the adult, but are self-renewing. However, although ependymal cells at the population level display cardinal stem cell features in vivo, such as self-renewal and generation of diverse progeny, it is difficult to study these properties at the single cell level in the tissue, and we cannot conclude that they act as stem cells in vivo.

In addition to ependymal cells, neural progenitors (expressing NG2, Olig2, and/or Nkx2.2) reside in the white and gray matter of the adult rodent spinal cord [6,43–46]. Different studies have suggested that the parenchymal progenitors represent multipotent neural stem cells or more-restricted glial progenitors [6,43,47]. Under the standard neurosphere assay conditions employed here, the vast majority of the neural stem cell potential resides within the ependymal population. However, we cannot exclude that other cells contribute, to a comparatively smaller degree, to neurosphere formation under our conditions or that they may display neural stem cell properties under other conditions. The parenchymal progenitors are likely to serve to replace glial cells in the uninjured spinal cord, which we do not find evidence that ependymal cells do. Parenchymal progenitors are rapidly depleted after spinal cord injury, but are later replaced and may participate in the generation of glial cells after injury [6,44,46]. It is possible that some of the ependyma-derived Olig2^+^ cells observed shortly after injury represent regenerated parenchymal progenitors.

The limited functional recovery typically associated with central nervous system injuries is in part due to the failure of severed axons to regrow and reinnervate their targets. Axonal regeneration is inhibited by scar formation and growth-inhibitory factors associated with myelin and astrocytes [48,49]. Modulating the responsiveness to axonal growth-inhibitory factors and glial scar formation are attractive strategies to improve functional recovery after central nervous system injuries [50–53]. The majority of ependyma-derived cells differentiate to astrocyte-like cells after injury and are found in the core of the scar tissue. However, these cells are found in complementary nonoverlapping domains to areas immunoreactive to CSPG, the most important axonal growth inhibitor associated with glial scars [35,36]. Moreover, axons in the scar tissue, most likely sprouts from severed axons growing into the scar tissue, were frequently found in direct proximity to ependyma-derived cells. This argues that ependyma-derived cells in the scar tissue do not constitute a major impediment to axonal growth, and may even suggest that they support some local sprouting.

Spinal cord injury results in the loss of oligodendrocytes and demyelination of axons even at some distance to the lesion [54,55]. Spinal cord injuries are most commonly incomplete in man, leaving spared tissue connecting the spinal cord above and below the lesion, but the function of remaining axons is often compromised due to demyelination. Without insulating sheaths of myelin, spared axons close to, but not directly affected by the injury, become less efficient in their ability to conduct electrical impulses [56]. Moreover, chronically demyelinated axons are vulnerable to degeneration. Axons are remyelinated with time, and this is thought to occur through the generation of new oligodendrocytes by stem or progenitor cells rather than by self-duplication of mature remaining oligodendrocytes [57–59]. We report here that ependymal cells contribute to the regeneration of oligodendrocytes and remyelination after spinal cord injury. The differentiation pattern of ependymal cells after injury is reminiscent to that seen for in vitro expanded adult spinal cord neural stem cells transplanted to the injured spinal cord [5]. Transplanted adult spinal cord-derived neurospheres improve functional recovery, and if they are forced to generate more oligodendrocytes, functional recovery is further improved [5]. Since ependymal cells are the main source of neurospheres from the adult spinal cord (Figure 3), promoting oligodendrocyte generation from these cells in vivo could potentially improve recovery after spinal cord injury. The development of pharmacological strategies to modulate endogenous stem cells and their progeny may be an attractive alternative to cell transplantation for the treatment of spinal cord injury.

## Materials and Methods

### Generation of transgenic mice.

For *Nestin-CreER*, we used the enhancer found in the second intron of the rat *nestin* gene fused to a minimal hsp68 promoter [18,60,61] that controls the expression of CreER^T2^ [62], as previously described [21]. For *FoxJ1-CreER*, we used a human FOXJ1 promoter [13] fused to a CreER^T2^ ires-EGFP construct. Transgenic mice were generated at the Karolinska Center for Transgene Technologies by standard procedures utilizing pronuclear injection of CBA × C57BL/six fertilized eggs. Potential founder animals were screened by Southern blot analysis and PCR analysis using a CreER^T2^-specific fragment as probe or PCR template. Founder mice were bred to wild-type C57Bl/6 mice. Expression of the transgene was analyzed by confocal microscopy of sections stained with anti-Cre antibodies and cell-specific markers. Recombination was induced by five daily intraperitoneal injections of 2 mg of tamoxifen (Sigma; 20 mg/ml in corn oil).

### Immunohistochemistry.

Adult mice were perfused transcardially with PBS followed by 4% formaldehyde in PBS, spinal cords were post-fixed overnight at 4 °C and then cryoprotected in 30% sucrose. Coronal (14 or 20 μm) or sagittal (20 μm, from ∼9-mm-long pieces) sections were collected alternating on ten slides (8–10 sections per slide). Sections were incubated with blocking solution (10% donkey serum in PBS, with 0.3% Triton-X100) for 1 h at room temperature, then incubated at 4 °C or room temperature in a humidified chamber for 12–48 h with primary antibodies diluted in blocking solution. For MBP staining, sections were first delipidized. For antibodies raised in mouse, the M.O.M. kit (Vector), ABC kit (Vector), and TSA system (Perkin Elmer) were used following the manufacturers' instructions. The following primary antibodies were used: β-galactosidase (1:5,000, rabbit; ICN Biomedicals, or 1:1,000, goat; Biogenesis), BrdU, (1:200, rat; Accurate), CD133 (1:500, rat, clone 13A4; eBioscience), chondroitin sulfate (1:1,000, mouse; Sigma), Cre (1:2,000, mouse; Nordic BioSite), Crocc (1:5,000, rabbit, Root6; gift from T.Li), CXCR4 (1:500, mouse; BD Pharmingen), fibronectin (1:1,000, rabbit; Sigma), GFAP (1:1,000, mouse, clone G-A-5; Sigma), Ki67 (1:1,000, rabbit; Neomarkers), Olig2 (1:500, goat; R&D Systems), laminin (1:1,000, rabbit; Sigma), MBP (1:500, rabbit; Chemicon), musashi-1 (1:2,000, rat, clone 14H1; gift from H.Okano), nestin (1:5,000, rabbit [63] or 1:500, mouse; BD Pharmingen), neurofilament heavy (1:1,000, chicken; Chemicon), PDGFRα (1:500; BD Pharmingen), RC1 (1:200, mouse; DSHB), Sox2 (1:500, mouse; Chemicon, or 1:1,000, goat; gift from J. Muhr), Sox3 (1:500, rabbit; gift from T.Edlund), Sox9 (1:500, goat; R&D Systems), vimentin (1:1,000, chicken; Chemicon). After washing, antibody staining was revealed using species-specific fluorophore-conjugated (Cy3, Cy5 from Jackson, and Alexa 488 from Molecular Probes) or biotin-conjugated secondary antibodies (Jackson). Biotinylated secondary antibodies were revealed using the ABC kit (Vector Labs) with TSA fluorescent amplification kit (Perkin-Elmer). Sections were counterstained with DAPI (1 μg/ml; Sigma). Control sections were stained with secondary antibody alone. Pictures were taken using a Zeiss Axioplan 2, Zeiss Axiovert 200M or a LSM510 META confocal microscope with Zeiss and Openlab (Improvision) software. Image processing and assembly was performed in ImageJ and Photoshop.

### Immunoelectron microscopy.

Anaesthetized mice were perfused transcardially with 4% paraformaldehyde in PBS. The spinal cord was dissected out and post-fixed for 4 h. Sections (60 μm) were immunolabled with Cre or β-gal antibodies in 0.1% Triton X100 and 10% donkey serum in PBS. A secondary antibody conjugated to biotin was used with an ABC kit (Vector Labs). In some sections, a fluorescent secondary antibody was used, and sections were labeled with DAPI to map the location of the nuclei of the surrounding cells (Figure S11). The sections were post-fixed in 3% glutaraldehyde in 0.1 M cacodylate buffer (pH 7.4) and in 1% osmium tetraoxide in 0.1 M cacodylate buffer, dehydrated in a graded series of ethanol, stained with uranyl acetate, and embedded in Durcupan resin (Fluka). Serial sections (200 nm) of *FoxJ1-CreER* spinal cord were used to define the ultrastructural morphology of cells lining the central canal. The complete series of sections from individual cells were traced in 90 sections to characterize the morphology in three dimensions. Serial semi- and ultrathin sections (2 μm and 70 nm, respectively) were used in correlative light and electron microscopic evaluation (CLEM) of the fate of ependyma-derived cells 4 wk after a spinal cord injury (Figure S6). The semithin sections were used to identify immunopositive cells, and the ultrathin sections to show the morphology of the identified cells. Sections (70–200 nm) were placed on Formvar-coated copper grids, counterstained with 2% uranyl acetate and Reynold's lead citrate. Sections were examined in a Tecnai 12 electron microscope (FEI) equipped with 2kx2k TemCam-F224HD camera (TVIPS).

### Neural stem cell cultures.

Spinal cords were dissected and cells dissociated using papain (Worthington). Neurospheres were cultured as described [8] in DMEM/F12 medium supplemented with B27 and EGF and bFGF (both 10 ng/ml). Approximately 200,000 cells were plated in 10-cm cultures dishes, corresponding to a density of 20 cells/microliter, which allows the generation of clonal neurospheres [64]. For assaying self-renewal and multipotentiality, *FoxJ1-CreER*x*R26R* (*n* = 6) and *Nestin-CreER*x*R26R* (*n* = 6) adult mice were administered with tamoxifen intraperitoneally for 5 d with a washout period of 6 d (see Figure 3). Single spheres were manually collected and split into two wells. One well was used for continuous passaging and subsequent neural stem cell differentiation, whereas the other well was used for X-gal staining. For assaying self-renewal, four clonal recombined neurospheres per animal were manually isolated after 12 d of primary neurosphere formation. All recombined neurospheres were serially passaged eight times. In vitro differentiation by growth factor withdrawal for 10 d was assessed in passage 3 and passage 6 by staining as described above for βIII-tubulin (Tuj1, 1:1,000; Covance), GFAP (1:5,000; DAKO), and O4 (1:200; Chemicon).

### Flow cytometry.

Spinal cords were dissected from *FoxJ1-CreER* mice and dissociated using papain (Worthington) and DNase in 1× HBSS at 37 °C for 1 h. Ovomucoid inhibitor (Worthington) was added and cells were collected by centrifugation at 300*g* for 5 min. Cells were resuspended in Leibovitz-15/B27 with 7AAD, which labels dead cells. Single GFP^+^ (based on the ires-GFP signal), 7AAD^−^ cells were isolated using a FACSAria (BD). Singlets were identified based on forward scatter width (FSC-W) versus forward scatter height (FCS-H) [65]. Single cell sorting and GFP fluorescence was confirmed by microscopic examination.

### Spinal cord injury, BrdU, and growth factor treatments.

Mice were anesthetized with 2.5% Avertin, and the dorsal funiculus at mid-thoracic level was cut transversely and was extended rostrally with microsurgical scissors to span one segment [39]. In other animals, the lateral funiculus was cut transversally and the lesion extended rostrally to span one segment.

BrdU (1 mg/ml and 1% sucrose, exchanged every 3–4 d) was administered in the drinking water to label dividing cells.

### Quantitative analyses.

In order to correlate recombination frequency in neurosphere cultures to the in vivo recombination frequency of spinal cord tissue, the ratio between CreER^+^ and β-gal^+^ cells (*n* = 60) was quantified in a small postfixed biopsy from the same spinal cords used for neurosphere cultures.

The percentage of BrdU^+^ ependymal cells was obtained from three animals treated for 4 wk with BrdU in the drinking water (3–5 coronal sections/animal analyzed). The total number of cells per section was obtained counting all nuclei stained with DAPI.

The in vivo recombination frequency was assessed by counting the number of recombined cells over the total number of ependymal cells (Vimentin^+^) from five coronal sections per animals at 2 d (4 animals: 2 *Nestin-CreERxR26R*, 2 *FoxJ1-CreERxR26R*) and 10 mo (3 animals: 3 *FoxJ1-CreERxR26R*) after tamoxifen treatment (Figure 4E).

The contribution of recombined cells at the site of injury was established by measuring the relative area occupied by β-gal^+^ cells within the epicenter of the lesion (using ImageJ software) of 3 *FoxJ1-CreER*x*R26R* animals (2 sagittal sections per animal) 2 wk after spinal cord injury.

The cell fate distribution of ependyma-derived progeny was obtained by scoring recombined cells positive for Olig2, GFAP or Sox9 in either coronal (3–5 sections per animal) or sagittal (1–2 sections per animal) sections encompassing the lesion site from 1 month (3 animals: 1 *Nestin-CreER*x*Z/EG*, 2 *FoxJ1-CreER*x*R26R*) and 8–10 mo (4 animals: 1 *Nestin-CreER*x*Z/EG* and 3 *FoxJ1-CreER*x*R26R*) after spinal cord injury (Figure 6M).

The frequency of proliferation of ependymal cells and their progeny was assessed by counting the number of Ki67^+^ recombined cells over the total number of recombined cells from three segments (rostral to, caudal to, and at the injury site; average of 15 coronal sections, or 300 recombined cells, per segment analyzed) from 2 *FoxJ1-CreERxR26R* animals 4 d after spinal cord injury (Figure S7C).

## Supporting Information

Figure S1 Schematic Illustration of Transgenic Constructs and Genetic Labeling Strategy (A) The *Nestin* 2nd intron central nervous system (CNS)-specific stem/progenitor enhancer (NestinE) with a minimal hsp68 promoter drives CreER expression. The human FoxJ1 promoter drives CreER and EGFP expression in ependymal cells with motile cilia. CreER protein is cytoplasmic due to the association with the heat-shock chaperone complex.
(B) Nuclear localization of CreER and recombination is induced upon 4-hydroxytamoxifen (4-OHT) binding to the ER domain. The reporter allele consists of a loxP-flanked transcriptional stop cassette. Cre recombination induces the expression of the reporter protein (β-gal or GFP) under a general and ubiquitous promoter.
(205 KB TIF)

Figure S2 Molecular Marker Expression by Adult Spinal Cord Ependymal Cells (A–D) Ciliary rootlet coiled-coil protein (Crocc, also known as Rootletin) is expressed in Sox2 (a neural stem cell marker) positive ciliated ependymal cells of the adult spinal cord and colocalizes with β-gal expression in recombined ependymal cells.
(E–H) Ependymal cells are immunoreactive to the neural stem cell-associated protein CD133/Prominin, which is localized at the luminal surface (shown in higher magnification in [H]).
(I–L) Nestin, but not GFAP, is expressed in recombined β-gal^+^ ependymal cells.
(M–P) The RC1 antigen associated with radial glial cells is expressed in ependymal cells and recombined ependymal cells express Sox9, an immature glial marker. The analysis is from uninjured *FoxJ1-CreERxR26R* mice 6 d after termination of tamoxifen administration. The scale bar indicates 25 μm.
(5.51 MB TIF)

Figure S3 Molecular Marker Expression by Adult Spinal Cord Ependymal Cells (A–D) Musashi-1 (Msi1), (E–H) PDGFRα and vimentin, all associated with neural stem/progenitor cells, are expressed in recombined ependymal cells. Recombined ependymal cells also express the neural stem cell-associated proteins Sox3 (I–L) and Sox2 (M–O), but not the astrocyte marker GFAP (D) nor the oligodendrocyte progenitor marker Olig2 (P). The analysis is from uninjured *FoxJ1-CreERxR26R* mice 6 d after termination of tamoxifen administration. The scale bar indicates 25 μm.
(6.78 MB TIF)

Figure S4 Correlative Light and Electron Microscopy (CLEM) Analysis (A) Schematic illustration showing the sequence of semithin and ultrathin sections used to study the morphology of cells migrating from the central canal in three dimensions. (B) An example of a β-gal^+^ cell identified at the light microscopic level following PAP immunocytochemistry. The labeled cell is shown in an electron micrograph from a neighboring, ultrathin section in (C). The scale bar indicates 2 μm.
(3.37 MB TIF)

Figure S5 Morphological Features of Ependymal Cells (A) An electron micrograph of ependymal cells (e) and tanycytes (t) lining the central canal of the spinal cord. Arrows point to processes extending from tanycytes. The inset shows a light micrograph of a neighboring 2-μm semithin section where the PAP staining is evident. Asterisks mark lipid droplets.
(B) An ependymal cell with a cilium (c) and multiple villi (v).
(C) An electron micrograph of a multiciliated tanycyte with a process. Note that the tanycyte has a darker cytoplasm compared to ependymal cells.
(D) A cross-section of a cilium with microtubules arranged in a 9 + 2 array. Only this type of cilium was found in ependymal cells, tanycytes, and radial ependymal cells.
(E) The electron dense PAP reaction product signaling for Cre is shown at high magnification.
(F) An example of an ependymal cell with two cilia. Arrows point to the basal bodies.
(G) A tight junction (arrow) between an ependymal cell and a tanycyte.
(H) An electron micrograph showing a Cre^+^ radial ependymal cell (r) with a process (arrow) and a lipid droplet (asterisk). The inset shows a light micrograph of the PAP labeling.
(I) High magnification of the cytoplasm and villi of a radial ependymal cell extending into the lumen of the central canal.
(J) An electron micrograph of the basal body and cilia of a radial ependymal cell.
(A–J) are from *FoxJ1-CreER* mice.
(K) Electron micrograph of Cre-immunopositive ependymal cells, radial ependymal cells, and tanycytes at the central canal of the spinal cord from the *Nestin-CreER* mouse.
bv: blood vessel; m: mitochondrion; n: nucleus.
The scale bars indicate 5 μm in (A) and (K), 1 μm in (B), (E), (F), and (J), 2 μm in (C) and (H), 0.1 μm in (D) and (G), and 0.25 μm in (I).
(7.38 MB TIF)

Figure S6 Exponential Growth of Ependymal Cell-Derived Neurosphere Cells In Vitro The number of cells obtained after sequential clonal passaging, starting with individual recombined primary neurospheres from *Nestin-CreER* crossed with R26REYFP mice [66]. Each line represents the mean ± SD from four clones from one animal.
(124 KB TIF)

Figure S7 Spinal Cord Injury Induces Proliferation of Ependymal Cells (A and B) Proliferation and Ki67 expression are induced in ependymal cells close to and in the injury (B), but not in adjacent uninjured segments (A).
(C) Quantification of the injury-induced proliferative response of ependymal cells at different locations relative to the epicenter of the injury.
(D–F) Representative image of the quantified area (D), with boxed areas depicting the central canal region (E) and the migrating ependyma-derived cells entering the lesion area (F).
Scale bars indicate 25 μm in (A), (B), (E), and (F), and 50 μm in (D).
(3.21 MB TIF)

Figure S8 Migration of Ependymal Cell Progeny to Lateral Spinal Cord Lesions (A–C) Distribution of β-gal–immunoreactive ependymal cells and their progeny 2 wk after a laminectomy only (sham [A]) and a right (B) or a left (C) lateral funiculus incision. GFAP immunoreactivity demarcates the injury site (indicated by hatched lines).
Four weeks after a lateral injury, ependymal cells give rise to Sox9^+^ (D), GFAP^+^ (E), and Olig2^+^ (F) cells, as after a dorsal funiculus lesion.
Scale bars indicate 200 μm in (A–C) and 20 μm in (D–F).
(2.22 MB TIF)

Figure S9 Relationship between Ependymal Cells, Their Progeny, and CXCR4 Immunoreactivity (A–F) Most ependymal cells (identified by β-gal and vimentin in a FoxJ1-CreERxR26R mouse) do not display detectable CXCR4-immunoreactivty (yellow arrowheads), although some ependymal cell processes appear CXCR4-immunoreactive (white arrowheads).
(G–I) Two weeks after a dorsal funiculus lesion, many cells in the forming scar tissue are CXCR4 immunoreactive, but the β-gal–expressing ependyma-derived cells appear negative for CXCR4.
Scale bars indicate 25 μm in (A–F) and 50 μm in (G–I).
(3.27 MB TIF)

Figure S10 Central Canal Ependymal Cells Are Not Depleted after Injury and Maintain Their Phenotype over 8 Mo (A and B) A sagittal section showing Sox9 and vimentin expression in recombined ependymal cells of the central canal in an uninjured segment 8 mo after spinal cord injury.
(C–E) In the injured segment of the same animal, ependyma-derived recombined (β-gal^+^) cells occupying the scar tissue ([C] and higher magnification in [D]) as well as ependymal cells at the central canal ([C] and higher magnification in [E]) are still Sox9^+^ and Vim^+^ 8 mo after injury.
Scale bars indicate 25 μm in (A) and (D) and 100 μm in (C) and (E).
(3.87 MB TIF)

Figure S11 Identification of an Ependyma-Derived Cell at the Ultrastructural Level (A and B) The nuclei of an ependyma-derived β-gal–immunoreactive cell and surrounding cells were labeled with DAPI. The β-gal–immunoreactive cell was identified in the electron microscope by the position of the nucleus in a 3D reconstruction of serial 120-nm-thick sections of the whole area.
(C) The β-gal–immunoreactive cell is indicated by an asterisk and surrounding cells are numbered. BV: blood vessel.
Scale bars indicate 10 μm.
(907 KB TIF)

Table S1 Morphological Characteristics of Ependymal Cells Details for the studied cells lining the central canal of the spinal cord by immunoelectron microscopy, which underlie classification into the cell types shown in Figure 1. Each cell shown in the table was traced in complete series of ultrathin sections. Color coding corresponds to the Venn diagram in Figure 1. Ependymal cells are cyan, tanycytes are orange, and radial ependymal cells are purple. Green represents an intermediate population of cells with a dark cytoplasm, without any process. Black represents a second intermediate population with a light cytoplasm, multiple cilia, and with a process. Red represents a population of cells with an irregular nucleus, light cytoplasm, a process, and only one cilium.
(323 KB TIF)

## Abbreviations

β-gal: β-galactosidase
BrdU: 5-bromo-2-deoxyuridine
CreER: tamoxifen-dependent Cre recombinase
CSPG: chondroitin sulphate proteoglycans
GFP: green fluorescent protein
GFAP: glial fibrillary acidic protein
MBP: myelin basic protein
SD: standard deviation
